# Evaluation of the pharmacokinetics and efficacy of transdermal flunixin for pain mitigation following castration in goats

**DOI:** 10.1093/tas/txaa198

**Published:** 2020-10-30

**Authors:** Meggan T Graves, Liesel Schneider, Sherry Cox, Marc Caldwell, Peter Krawczel, Amanda Lee, Andrea Lear

**Affiliations:** 1 Large Animal Clinical Sciences, University of Tennessee College of Veterinary Medicine, Knoxville, TN; 2 Biomedical and Diagnostic Sciences, University of Tennessee College of Veterinary Medicine, Knoxville, TN; 3 Department of Animal Science, University of Tennessee Institute of Agriculture, Knoxville, TN; 4 Department of Agricultural Sciences and Animal Science, University of Helsinki, Helsinki, Finland; 5 Department of Production Animal Medicine at the Research Centre for Animal Welfare, University of Helsinki, Helsinki, Finland; 6 Helsinki One Health (HOH), University of Helsinki, Helsinki, Finland

**Keywords:** castration, flunixin, goat, pain, transdermal

## Abstract

The mitigation of pain associated with common management procedures is a rising concern among veterinarians, producers and consumers. Nonsteroidal anti-inflammatory drugs are vital compounds for this purpose due to their cost, convenience, and efficacy. A transdermal formulation of flunixin meglumine (FM) was approved for the treatment of pain in cattle; however, the efficacy has yet to be determined for small ruminants. The current study had two aims: 1) to determine the pharmacokinetics of transdermal flunixin meglumine (TD FM) in bucklings and 2) to evaluate pain mitigation by TD FM following castration. To evaluate pharmacokinetics, 12 male goats (mean age = 6 mo) received 2.2 mg/kg of FM IV (*n* = 6) or 3.3 mg/kg TD FM (*n* = 6). Plasma FM concentrations were measured. The mean *C*_max_, *T*_max_, and harmonic mean half-life for TD FM were 1.09 ± 0.65 μg/mL, 5.50 ± 2.95 h, and 7.16 ± 2.06 h, respectively. To evaluate the efficacy of pain mitigation, 18 goats were randomly assigned to three treatment groups: 1) TD FM and castration (FM CAST) (*n* = 6); 2) transdermal placebo and castration (PL CAST) (*n* = 6); and 3) TD FM and sham castration (SHAM) (*n* = 6). Plasma samples were collected at 0, 12, 24, 36, 48, 72, and 96 h to assess cortisol and prostaglandin E_2_ (PGE_2_)_._ Daily dry matter intake (DMI) was recorded and body weight was measured at the beginning and end of the study. Thermography (IRT) images of the scrotum, as well as heart rate (HR), respiratory rate (RR), and rectal temperature, were taken twice daily. Separate mixed analysis of variance models were used to test the effects of treatment, time, and their interaction on mean body temperature, IRT, HR, and RR. Autoregressive covariance structure was utilized to account for repeated measures and individual goat DMI prior to the study was added as a covariate. There were no differences in vital parameters, IRT measurements, cortisol, or PGE_2_ in animals receiving either TD FM or placebo following castration (*P* > 0.05). DMI had a treatment by hour interaction and was significantly higher in FM CAST and SHAM groups than the PL CAST group (*P* = 0.04). Goats in the SHAM group gained weight throughout the study, whereas goats in all other groups lost weight (*P* = 0.02). Results indicate that TD FM may mitigate pain as demonstrated by increased DMI; however, a single dose may not be sufficient to reduce physiological indicators of pain associated with castration in goats.

## INTRODUCTION

Routine management practices, such as castration, produce acute stress, and discomfort, which can lead to reduced performance and compromised animal well-being (Greenwood and Shutt, 1990; Ahmed, 2011; Alvarez et al., 2015). As the consuming public becomes more aware of production methods, it will be vital for producers and veterinarians to have access to validated therapeutic information in order to make judicious pain management decisions to alleviate suffering and improve animal welfare (Bowen and Snyder, 2009). Pain caused by necessary management practices can be reduced with analgesics and anti-inflammatories. A transdermal application of flunixin meglumine (FM), a common nonsteroidal anti-inflammatory drug (NSAID), has been registered for use in cattle and shown to successfully reduce both fever and inflammation in calves with respiratory disease (Thiry et al., 2015). However, it may not provide significant analgesic effects following painful stimuli, as demonstrated following dehorning and castration in calves (Kleinhenz et al., 2017; Kleinhenz et al., 2018b).

Due to the lack of small ruminant specific therapeutics, the objective of this study was to identify whether or not transdermal flunixin meglumine (TD FM) is beneficial for goats by determining the pharmacokinetics of TD FM in young male goats and the efficacy of transdermal application on pain mitigation postcastration in comparison to goats receiving no NSAID administration. The investigators hypothesized that TD FM would reach measurable concentrations in goats following administration as demonstrated previously by Reppert et al (2019). Considering the pharmacokinetics of other topically applied drugs in goats compared to cattle, the researchers anticipated reduced maximum plasma concentrations as compared to bovine. The second hypothesis was that goats treated with TD FM would demonstrate higher dry matter intake (DMI); reduced scrotal temperatures when evaluated with infrared thermography imaging (IRT); and reduced plasma concentrations of stress and inflammatory biomarkers compared to placebo-treated controls.

## MATERIALS AND METHODS

This study was approved by the Institutional Animal Care and Use Committee at the University of Tennessee (IACUC # 2563-1017).

### Animal Acquisition

Eighteen intact male mix breed goats with a mean weight of 26.4 ±1.6 kg were obtained for the study carried out in the late spring months of May and June 2019. All goats were examined at purchase and found to be clinically healthy and without fever. Goats were administered metaphylactic antibiotics (tulathromycin 100 mg/mL, 2.2 mg/kg SQ; Draxxin, Zoetis Inc., Parsippany, NJ), dewormed, according to manufacturer’s instructions, following acquisition (sulfadimethoxine oral suspension 5%, Albon, Zoetis Inc., Parsippany, NJ), moxidectin 1 mg/mL, Cydectin Oral Sheep Drench, Zoetis Inc., Parsippany, NJ), and allowed two weeks of pasture access, as well as fed hay and goat pellets twice daily prior to the start of the study to facilitate visual health screenings.

### Experiment 1: Pharmacokinetic Determination of TD FM

A completely randomized design was implemented in which 12 goats were selected to participate and randomly assigned to one of two treatment groups. All goats were placed in individual pens approximately 1.52 m × 2.13 m in size in a well-ventilated barn. Twenty-four hours prior to the start of the study, the hair was clipped over both jugular veins using a #40 blade and cordless clippers (Oster PowerPro). The goats were restrained with halters, and 16 gauge catheters were aseptically placed in the right jugular vein of each goat. At time 0 h (prior to treatment), 2 mL of blood were collected in a heparinized tube (BD Vacutainer) and placed on ice. Goats in group one then immediately received a single application of TD FM (Banamine Transdermal, Merck Animal Heath, Intervet Inc.) at 3.3 mg/kg (65 to 90 mg) applied via a single-use syringe to the skin along the dorsal midline from the shoulders to the tail head. Immediately following 0 h sample collection, goats in group two received a single intravenous dose of FM at 2.2 mg/kg via percutaneous needle injection of the left jugular vein. Additional blood samples were collected individually at 3, 5, 10, 15, and 30 min and 1, 2, 4, 8, 12, 24, 36, 48, 60, 72, 84, and 96 h post-treatment. All goats remained haltered and tied for the first 5 post-administration sample collections (3, 5, 10, 15, and 30 min). Goats were caught and haltered 5 min prior to subsequent sample time points. Preceding each sample collection, the injection port was disinfected with an alcohol swab and a blood volume equivalent to the catheter and extension set was discarded to prevent dilution with heparinized flush. Samples were placed on ice and centrifuged within 1 h of collection at 2000 × *g* for 15 min. Plasma was transferred to cryovials for storage at −80 °C until analyzed.

### Plasma Flunixin Determination

Analysis of flunixin in plasma samples was conducted using reversed phase high pressure liquid chromatography (HPLC). The system consisted of a 2695 separations module and a 2487 UV detector (Waters, Milford, MA, USA). Separation was attained on a Waters XBridge C18 4.6 × 100 mm (3.5 µm) protected by a 3.5 µm XBridge C18 guard column. The mobile phase was an isocratic mixture of 0.1% formic acid in water pH 3.0 with 1 M NaOH and acetonitrile (58:42). The mixture was prepared fresh daily using double-distilled, deionized water filtered (0.22 μm) and degassed before use. The flow rate was 1.0 mL/min and UV absorbance was measured at 280 nm.

Flunixin was extracted from plasma samples using a liquid–liquid extraction. Briefly, previously frozen plasma samples were thawed and vortexed and 100 µL was transferred to a screw-top test tube followed by 25 µL internal standard (10 µg/mL meloxicam). One hundred microliters of 1 M HCl was added followed by 2 mL of chloroform and the tubes were vortexed for 30 s and then centrifuged for 10 min at 1000 × *g*. The organic layer was transferred to a 16 × 100 mm tube and evaporated to dryness with nitrogen gas. Samples were reconstituted in 250 µL of mobile phase and 100 µL was analyzed.

Standard curves for plasma analysis were prepared by fortifying untreated, pooled goat plasma with flunixin to produce a linear concentration range of 5 to 50,000 ng/mL. Calibration samples were prepared exactly as plasma samples. Average recovery for flunixin was 93%, while intra- and inter-assay variability ranged from 2.5% to 6.3% and 4.4% to 7.7%, respectively. The lower limit of quantification was 5 ng/mL.

### Pharmacokinetic Analysis

Plasma flunixin concentrations were analyzed for each individual animal by compartmental and noncompartmental approaches using Phoenix 7.0 (Certara, St. Louis, MO). The TD FM data was analyzed using noncompartmental analysis while the IV data was analyzed using compartmental and noncompartmental analysis. Biexponential equations for a two-compartment model were used to fit the IV data using the equation:

$$\begin{array}{l} Cp\;=\;Ae-\alpha t\;+\;Be-\beta t \end{array}$$

*A* and *B* indicate *y*-intercept constants, *α* is the distribution rate constant and *β* is the elimination rate constant.

Weighting of the data using the 1/(Yhat × Yhat) of the concentration was used to improve the line fit and residual plots. The goodness of fit of the data with the model was determined by visual examination of the line fits, residual plots, and Akaike’s information criteria (Yamakoa et al., 1978). Values for elimination rate constant (*λ*_z_), plasma half-life (*t*_½_), maximum plasma concentration (*C*_max_), time to maximum plasma concentration (*T*_max_), apparent volume of distribution (*V*_darea_), apparent volume of distribution at steady state (*V*_dss_), total body clearance (Cl), and area under the plasma concentration time curve (AUC_0–∞_) from time 0 h to infinity were calculated from noncompartmental analysis (Tables 1 and 2). The AUC and AUMC were calculated using the log-linear trapezoidal rule. Mean residence time (MRT) was calculated as $AUMC_{0-\infty }/AUC_{0-\infty }.$ Parameter values were reported for each individual animal.

**Table 1.** Plasma pharmacokinetic parameters of intravenous (2.2 mg/kg) administered flunixin in six goats

Pharmacokinetic parameter	Goat 61	Goat 63	Goat 66	Goat 67	Goat 68	Goat 70	Mean ± SD
*t*_½_, h	4.80	3.49	4.13	4.24	1.88	4.18	3.79 ± 1.02
λ _z_, 1/h	0.14	0.20	0.17	0.16	0.37	0.17	0.20 ± 0.08
C_0_, μg/mL	43.33	33.17	37.54	122.86	35.25	32.31	50.74 ± 35.55
Cl, mL/h/kg	87.68	89.41	77.47	62.06	147.53	64.79	88.16 ± 31.20
*V*_dss_, mL/kg	218	226	218	195	127	236	203 ± 40
*V*_d(area)_, mL/kg	607	451	462	380	399	391	448 ± 85
AUC_0–∞_, h∙µg/mL	25.09	24.61	28.40	35.45	14.91	33.96	27.07 ± 7.45
AUC_Extrap_, %	0.88	0.37	0.65	0.16	0.15	0.16	0.39 ± 0.31
MRT_0–∞_, h	2.48	2.52	2.81	3.15	0.86	3.65	2.58 ± 0.95
*A*, µg/mL	34.01	25.12	36.02	41.52	37.80	33.52	34.66 ± 5.51
*B*, µg/mL	1.13	2.17	1.99	2.32	1.28	3.11	2.00 ± 0.73
*α*, 1/h	2.11	2.03	2.41	2.54	3.60	2.58	2.54 ± 0.56
*β*, 1/ h	0.15	0.20	0.17	0.15	0.37	0.16	0.20 ± 0.09
*t*_½_ α, h	0.33	0.34	0.29	0.27	0.19	0.27	0.28 ± 0.05
*t*_½_ β, h	4.74	3.49	4.08	4.55	1.87	4.24	3.83 ± 1.05

Terminal half-life; *t*_½_, plasma half-life; *λ*_z_, elimination rate constant; *C*_0_, estimated initial drug concentration in plasma; Cl, total body clearance; *V*_darea_, volume of distribution; *V*_dss_, apparent volume of distribution at steady-state; AUC_0–∞_, area under the plasma concentration time curve from time 0 to infinity; AUC_Extrap_, percent of the AUC_0–∞_ extrapolated to infinity, MRT_0–∞_, mean residence time; *A*, distribution intercept; *B*, elimination intercept; *α*, distribution constant; *β*, elimination constant; *t*_½_*α*, distribution half-life; *t*_½_*β*, elimination half-life.

**Table 2.** Plasma pharmacokinetic parameters of transdermal (3.3 mg/kg) administered flunixin in six goats

Pharmacokinetic parameter	Goat 53	Goat 54	Goat 56	Goat 57	Goat 58	Goat 60	Mean ± SD
*t*_½_, h	5.07	9.00	5.89	8.36	6.78	10.83	7.65 ± 2.14
*λ*_z_, 1/h	0.14	0.08	0.12	0.08	0.10	0.06	0.10 ± 0.03
*T*_max_, h	8	4	1	8	4	8	5.5 ± 2.9
*C*_max_, µg/mL	0.93	0.55	1.44	2.25	0.59	0.80	1.09 ± 0.65
AUC_0–∞_, h∙µg/mL	12.37	7.68	14.70	28.42	7.29	15.73	14.36 ± 7.72
AUC_Extrap_, %	0.71	2.03	0.40	0.30	2.55	0.50	1.08 ± 0.96
MRT_0–∞_, h	10.71	10.95	8.12	11.47	11.19	18.62	11.84 ± 3.53

Terminal half-life; *t*_½_, plasma half-life; *λ*_z_, elimination rate constant; *C*_max_, maximum plasma concentration; *T*_max_, time to maximum plasma concentration; AUC_0–∞_, area under the plasma concentration time curve from time 0 to infinity; AUC_Extrap_, percent of the AUC_0–∞_ extrapolated to infinity, MRT_0–∞_, mean residence time.

### Experiment 2: Castration and Pain Mitigation

**Figure 1.**
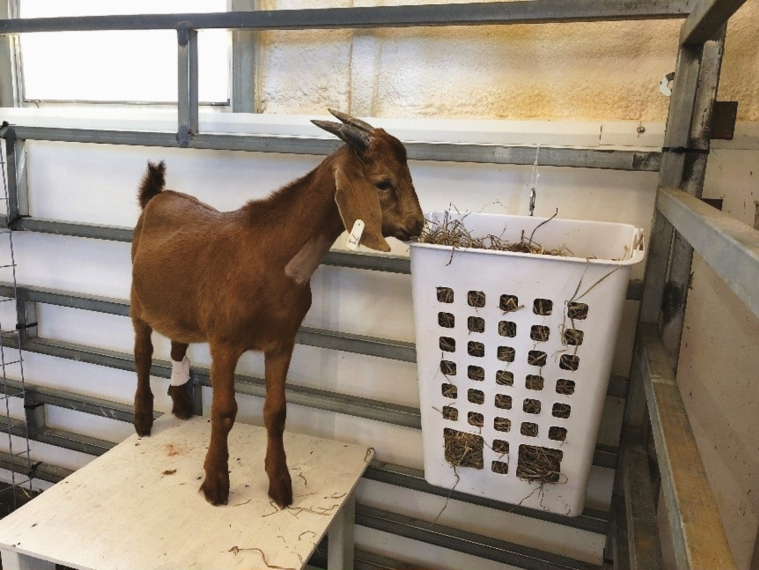
Image of goat in individual housing consisting of 1.52 m × 2.13 m pen with wood climbing box (0.6 m × 0.6 m × 0.46 m) and suspended hay basket.

Goats were returned to pasture for a four week washout period following experiment 1. At the completion of the washout period the following pilot study was performed. A completely randomized, single-blinded design was implemented, in which 18 goats were randomly assigned (using a random number generator) to each of three treatment groups: 1) transdermal flunixin and surgical castration (FM CAST; *n* = 6); 2) transdermal placebo and surgical castration (PL CAST; *n* = 6); and 3) TD FM and sham castration (SHAM; *n* = 6). All goats were housed individually and allowed two days to acclimate to pens prior to any treatment. Pens were approximately 1.52 m × 2.13 m in size. Each pen included a wood climbing box of 0.6 m × 0.6 m × 0.46 m dimension, free choice hay and water, and a mineral block (Figure 1).

### Body Weight

All goats were weighed individually on a single scale one day prior to the start of the study and again at the completion of the study (144 h).

### Dry Matter Intake

Mixed grass hay was fed free-choice at all times in suspended baskets (Figure 1). All baskets were weighed on the same neonatal scale twice daily before and after hay was added. Pellets were discontinued during experiment 2. Weights were recorded 2 days prior to castration and daily for 6 days following.

### Heart rate, Respiratory Rate, and Body Temperature

Vital parameters were recorded twice daily (between 6:00 to 8:00 a.m. and 18:00 to 20:00 p.m.) beginning 24 h prior to castrations and continuing for 6 days following. Heart rate (HR) was measured via use of a stethoscope. Respiratory rate (RR) was counted by observation of chest wall. A digital thermometer was utilized to acquire body temperature. Daily ambient temperatures were obtained from an official weather station within 5 miles from the study site with minimum and maximum temperature correlating with morning and evening data collection time points.

### Castration Technique

Castrations were performed on goats in a clockwise manner through the barn in which they were individually housed; housing arrangement was also randomized. Goats in all groups were sedated with 0.15 mg/kg of xylazine intramuscularly to produce lateral recumbency. For goats in groups 1 and 2 (FM CAST and PL CAST, respectively), the rear leg was elevated and secured with a rope and a subcutaneous ring block of 1% lidocaine was injected around the neck of the scrotum and into the tissue of each spermatic cord with a maximum volume of 2 mL. The scrotal skin was aseptically prepped with betadine and alcohol soaked gauze. Surgical castrations were performed by the standard technique consisting of removal of the distal half of the scrotum with a #10 scalpel blade by horizontal circumferential incision, followed by manually isolating each testis and freeing the fascia and connective tissue. The spermatic vessels were ligated with transfixing and circumferential sutures with 2–0 PDS prior to removing the testicle. Each surgery was performed by the same surgeon and took a maximum of 10 min. Goats in group 3 received a SHAM castration in which all steps were mimicked, but not performed, for the same average overall time as the routine surgical castrations of groups 1 and 2.

### Transdermal Flunixin and Placebo Application

Approximately 2 to 5 min following castration, while goats were still sedated, intravenous jugular catheters were placed aseptically as described in phase one. Time 0 h blood samples were collected in heparinized blood tubes (BD Vacutainer) immediately after placement of catheters and placed on ice. Goats were then given their assigned treatment. FM CAST and SHAM goats received 3.3 mg/kg TD FM (Banamine Transdermal, Merck Animal Heath, Intervet Inc.) as a single application along their top-line from the withers to the tail head. PL CAST goats received a topical placebo consisting of propylene glycol, isopropyl alcohol, and red dye to mimic the TD FM product of equal volume to the TD FM dose.

The application of the treatment or placebo for each respective group was considered time 0 h and the initiation of the experiment. A single unblinded individual, who was not involved in biologic sample analysis or thermography, carried out administration of the treatment or placebo. All other research personnel involved in the castrations and sampling were blinded to the contents of each syringe, and, therefore, the treatment allocation of each animal, until the end of the data collection phase of this project.

### Cortisol and PGE_2_ ELISA Sampling

In addition to hour 0, venous blood was collected via intravenous catheter at 12, 24, 48, 72, and 96 h and transferred to heparinized tubes. Samples were stored on ice until centrifuged at 2000 × *g* for 15 min within 1 h of collection. Plasma was transferred by pipette to cryovials for storage at −80 °C until analyzed. Plasma samples from each time point were analyzed for cortisol and PGE_2_ concentrations. Analysis of each biomarker was performed by the investigators through commercially available ELISA assays validated on caprine plasma, respectively (MyBioSource, San Diego, CA). Samples were run in duplicate. The cortisol assay had a detection range of 5 to 160 ng/mL with a coefficient of variation for intra-assay variability of <10% and inter-assay of <15%. The detection range for the goat PGE_2_ ELISA assay was 15.6 to 1000 pg/mL with intra-assay precision of ≤8% and inter-assay of ≤12%.

### Thermography

IRT of the scrotum was performed 24 h prior to castration and repeated twice daily for 7 days using an HD thermography unit (Fluke VT04A Visual IR Thermometer, Fluke Corporation, Everett, WA) with a temperature range of −20 to +250 °C and minimum focus distance of 25 cm. The camera is equipped with automatic internal calibration to account for changes in ambient temperature. Scrotal temperature was measured with the researcher standing approximately 1.0 m away with the focal point on the base of the scrotum. Images were saved on a micro SD memory card and uploaded to a laptop at the end of each day. Images were analyzed using computer software (Smartview Ver 7.0, Fluke Corporation, Everett, WA) and temperatures recorded.

### Statistical Analysis

Power analysis was conducted to estimate the sample size for detecting the difference in pharmacokinetics of transdermal flunixin compared to intravenous flunixin meglumine in healthy goats (experiment 1). In addition, experiment two looked at several biologic parameters and physical parameters throughout the study and the following were used for power analysis: plasma cortisol levels, plasma haptoglobin levels, plasma prostaglandin levels, heart rate, dry matter intake, infrared thermography, and a visual analog of pain. The preliminary information for power analysis was obtained from previous literatures on similar research (Mellor and Molony, 1991; Proudfoot et al., 2009; Thiry et al., 2017; Kleinhenz et al., 2018b). Power analysis was conducted using PROC GLMPOWER in SAS9.4 TS1M3 for Windows 64× (SAS Institute, Cary, NC). Power analysis showed that four to six animals per group were needed for detecting the group effect with 80% of power for a significance level of 0.05. This estimated sample size is consistent with the previous literature for the similar studies (Alvinerie et al., 1999; Königsson et al., 2003; Kleinhenz et al., 2018b; Straticò et al., 2018).

Prior to conducting statistical analyses, we removed one goat (ID # 56) which belonged to the FM CAST treatment from the study due to health issues unrelated to the purpose of the study. All statistical analyses were performed using SAS v 9.4 (Cary, NC). Outcomes of interest included HR, RR, body temperature, IRT, DMI, and weight gain. The correlation procedure was used to determine the relationships between body temperature, scrotal temperature and ambient temperature for animals within each treatment group using the “by treatment” option. To test the effect of treatment, time, and their interaction on mean body temperature, IRT, HR, and RR, separate mixed model analyses of variance (PROC GLIMMIX) were conducted, and the random effect of goat was included in all models. Autoregressive type one covariance structure was utilized to account for repeated measures collected over time from each goat subject. When analyzing the effects of treatment, time, and their interaction on DMI, each goat’s average pre-study (2 days prior to the administration of treatments) DMI was added into the model as a covariate. For all analyses, statistical significance was determined at *P* ≤ 0.05.

## RESULTS AND DISCUSSION

### Objective 1: Pharmacokinetic Analysis

**Figure 2.**
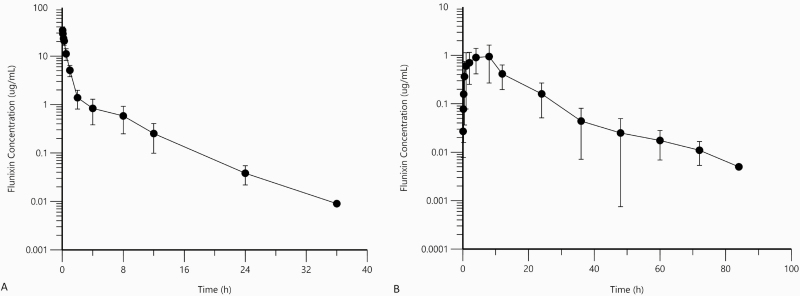
Mean ± SD plasma flunixin concentrations (A) following intravenous administration of flunixin (2.2 mg/kg; *n* = 6) and (B) transdermal administration of flunixin (3.3 mg/kg; *n* = 6) in independent treatment groups. Plasma samples were collected between 0 (baseline) and 36 h for the IV flunixin treatment group and between 0 and 96 h for the transdermal flunixin treatment group.

No adverse effects were observed following intravenous or TD FM administration. Mean standard deviation plasma concentrations for intravenous FM (Figure 2A) and TD FM (Figure 2B) were plotted and pharmacokinetic parameters reported in Tables 1 and 2. The mean *C*_max_, *T*_max_, and harmonic mean half-life for flunixin following transdermal application were 1.09 ± 0.65 μg/mL, 5.50 ± 2.95 h, and 7.16 ± 2.06 h, respectively.

TD FM is rapidly absorbed in cattle, with a longer half-life compared to intravenous use. The mean bioavailability of topical FM in cattle is 48% and the mean half-life of topical FM in cattle is 6.42 h (Kleinhenz et al., 2016). A recent study by Reppert et al. (2019) reported TD FM pharmacokinetic data for adult female goats, but there have been no studies evaluating TD FM in young male goats. Oral, intramuscular, and intravenous pharmacokinetic data have also been reported for adult goats suggesting that the intravenous dose of 2.2 mg/kg is applicable for goats (Konigsson et al., 2003). Other pour-on products such as topical eprinomectin have reported lower bioavailability in goats than in cattle (Rehbein et al., 2014). Eprinomectin has a much lower plasma concentration in goats compared to cattle designated by lower *C*_max_ and AUC indicating the recommended bovine dosage may not be adequate in caprine (Alvinerie et al., 1999).

The *V*_dss_ of 203 ± 40 mL/kg for IV FM found in the current study was lower than that reported in adult goats by both Reppert et al. (2019) and Konigsson et al. (2003), 303.6 mL/kg and 350.0 mL/kg, respectively. The terminal half-life found in the current study, 3.44 ±1.65 h, was comparable to that found by Konigsson et al. (2003), 3.6 h.

The AUC, 14.36 ±7.72 h∙μg/mL, following TD FM application was higher than that reported in adult goats, alpacas and dairy cattle, 12.18, 6.315, and 10.68 h∙μg/mL, respectively (Kleinhenz et al., 2016; Reppert et al., 2019a, 2019b). The *C*_max_ for TD FM (1.09 ± 0.65 μg/mL) found in this study was similar to that reported previously in calves (1.17 μg/mL) and for oral FM administration (1.2 μg/mL) of a 2.2 mg/kg dose (Königsson et al., 2003; Kleinhenz et al., 2016).

### Objective 2: Body Weight

There were no differences in pre-study (*P* = 0.95) or post-study (*P* = 0.35) body weight between FM CAST (Pre: 26.3 ± 1.6 kg; Post: 24.5 ± 1.5 kg), PL CAST (Pre: 26.2 ± 1.6 kg; Post: 24.5 ± 1.5 kg) or SHAM (Pre: 26.9 ± 1.6 kg; Post: 27.3 ± 1.5 kg) treatment groups (Table 3). There was a statistical difference (*P* = 0.02) in body weight gain during the study, where goats in the SHAM group gained 0.47 kg (±0.56), and goats in FM CAST and PL CAST groups lost 1.87 kg (±0.56) and 1.7 kg (±0.56), respectively. Presumed stress from castration has been documented to lead to lower average daily gain for one month following castration using the Burdizo method when compared with intact goats (Zamiri et al., 2012). Zamiri et al. documented a weight loss of 2 kg 20 days following castration when compared to intact goats, which is similar to the current study. Loss of average daily gain has also been documented in calves following surgical castration with local anesthesia for the week succeeding castration (Fisher et al., 1996). The data from the current study suggests that a single dose of TD FM is not sufficient to mitigate the predicted weight loss associated with castration. Daily weights were not measured on animals, presenting a limitation of this study. There may have been daily differences not recorded in light of the DMI data as one study in feedlot cattle showed increased average daily gain in castrates receiving multimodal analgesia (Repenning et al., 2013). Future research is warranted to investigate average daily gain differences following castration in goats receiving flunixin.

**Table 3.** Treatment means for body temperature, scrotal thermography, heart rate, respiratory rate, serum cortisol and PGE_2_, and body weight gain for each treatment group (PL CAST = TD placebo + castration; FM CAST = TD FM + castration; SHAM = TD FM + sham castration). Treatment means with different superscript letters are statistically different at *α* = 0.05 for both hour and/or treatment

			Treatment Means (± SE)		
Response	Hour *P-*value	Treatment *P-*value	PL CAST (*n* = 6)	FM CAST (*n* = 6)	SHAM (*n* = 6)
Body temperature, °C	<0.0001	0.03	39.36^a^ (±0.089)	39.33^a^ (±0.098)	39.00^b^ (±0.089)
Thermography, °C	<0.0001	0.03	34.58^a^ (±0.42)	34.88^a^ (±0.46)	33.21^b^ (±0.42)
Heart rate	0.003	0.12	113.3^a^ (± 5.8)	107.9^a^ (± 6.3)	95.2^a^ (± 5.8)
Respiration rate	0.10	0.36	36.3^a^ (± 1.9)	34.6^a^ (± 2.1)	32.1^a^ (± 1.9)
Serum cortisol	0.29	0.87	297.9^a^ (± 160.2)	456.6^a^ (± 160.2)	340.1^a^ (± 160.2)
PGE_2_	0.80	0.17	59.4^a^ (± 38.4)	133.1^a^ (± 34.8)	32.5^a^ (± 33.3)
Mean weight gain, kg		0.018	−1.7^a^ (±.056)	−1.87^a^ (±0.56)	0.47^b^ (±0.56)

Superscript letters a and b are meant to denote statistical differences.

### Dry Matter Intake

**Figure 3.**
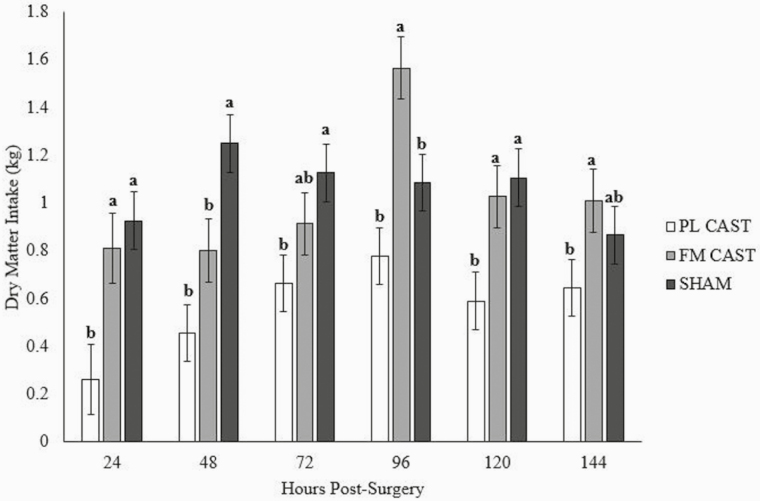
A treatment by hour postsurgery interaction was observed with respect to dry matter intake (*P* = 0.04). Dry matter intake (DMI) was calculated daily and displayed as means by treatment. Within each hour, columns with superscripts designate significant differences. PL CAST, transdermal placebo and castration (*n* = 6); FM CAST, transdermal flunixin meglumine (3.3 mg/kg) and castration (*n* = 6); SHAM, transdermal flunixin meglumine (3.3 mg/kg) and sham castration (*n* = 6).

After accounting for the covariate of individual average DMI during the pre-study period, DMI was associated with a treatment by hour interaction (*P* = 0.04) (Figure 3). When looking at treatment differences within each time-point, treatment effects varied across time. Although both PL CAST and FM CAST animals consumed less in the 24 h period following surgery as compared to the SHAM group, the FM CAST group did show a positive statistical difference when compared to the PL CAST group at all data collection points other than 48 h. This indicates that TD FM could alleviate pain, allowing animals to stand and eat more often. Other than at 48 h, the FM CAST group consumed the same amount or more than the SHAM group; however, this did not correlate with an overall weight gain as previously stated. Calves have been reported to have similar average daily feed intake following surgical castration with local anesthesia when compared to control calves for the first 5 days post castration; however, these results were reported for a 0- to 5-day period and not daily. A negative weight gain at one week postcastration was still noted in these animals as well (Fisher et al., 1996). Despite the increased hay consumption, FM CAST animals still had to address the increased energy demands associated with wound healing. This could explain why they still lost weight over the course of the study. Additionally, FM CAST goats were observed to be more active than PL CAST animals, perhaps accounting for decreased weight gain in the presence of increased hay intake.

### Body Temperature

**Figure 4.**
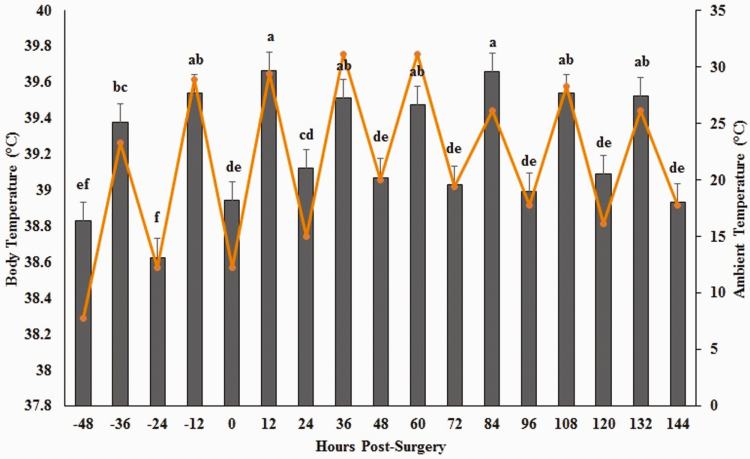
Least square mean body temperature for all animals in reference to ambient temperature. Body temperature is noted with gray bars, and ambient temperatures with orange line. Superscripts designate significance at *α* = 0.05.

Within all treatment groups, ambient temperature was significantly and positively correlated with body temperature (*ρ* = 0.52, *P <* 0.0001). Mean body temperature differed due to the main effects of treatment (*P =* 0.03) and hour (*P* < 0.0001) (Figure 4). There was no interaction between treatment and time (*P* = 0.07). Mean body temperatures were not different between animals in the PL CAST and FM CAST treatment groups, but SHAM animals exhibited significantly lower body temperatures throughout the study (Table 3).

### Scrotal Temperatures

**Figure 5.**
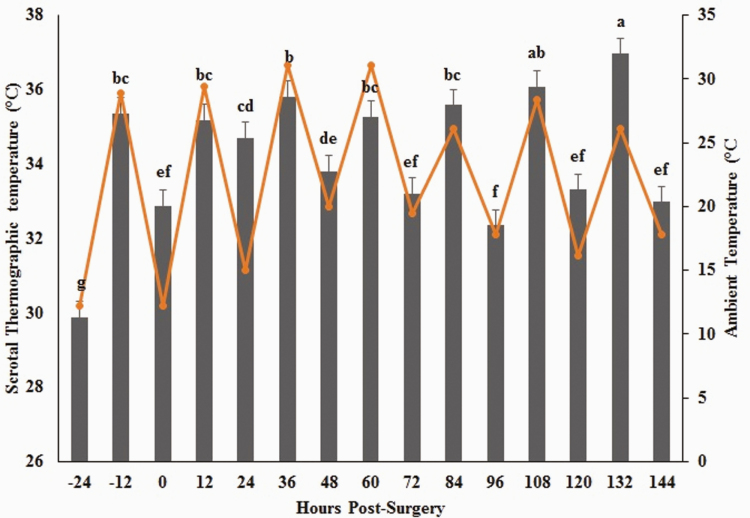
Least square mean scrotal temperature for all animals in reference to ambient temperature. Body temperature is noted with gray bars, and ambient temperatures with orange line. Columns with different letter groups are statistically different at *α* = 0.05.

Within all treatment groups, ambient temperature was significantly and positively correlated with scrotal temperature (*ρ* = 0.56, *P <* 0.0001) (Figure 5). There were similar strength, positive correlations between scrotal and body temperatures in the FM CAST (*ρ* = 0.57, *P <* 0.0001) and SHAM (*ρ* = 0.6, *P <* 0.0001) groups, respectively. Interestingly, in the PL CAST group, there was no relationship between body temperature and scrotal temperature (*ρ* = 0.18, *P* = 0.10). Mean scrotal temperature as measured by IRT differed due to the main effects of treatment (*P* = 0.03) and hour (*P* < 0.0001).

SHAM animals exhibited significantly lower scrotal temperatures compared to either PL CAST or FM CAST groups (*P* < 0.0001) (Table 3). Other studies have recorded lower scrotal temperatures for castrated animals in comparison to control animals when scrotal temperatures are measured within the first 120 min following surgery, possibly reflecting the disruption of blood supply indicating that higher scrotal temperatures in castrated animals is likely inflammatory related (Meléndez et al., 2017). Infrared thermography has been shown to detect inflammatory processes and heat stress in testicular studies of cattle and sheep (Cruz Júnior et al., 2015; Marti et al., 2017; Menegassi et al., 2018). These results indicate that a single dose of TD FM is not adequate to reduce castration-associated inflammation as measured by thermography.

### Heart Rate and Respiration Rate

There was no main effect of treatment (*P* = 0.12) or treatment by time interaction on mean HR response (*P* = 0.43) (Table 3). There was, however, an effect of time on HR that correlates with time of day and temperature. Respiration rate was not affected by treatment (*P* = 0.36), time (*P* = 0.10) or a treatment by time interaction (*P* = 0.26). Straticò et al. (2018) documented an elevation of HR and RR in lambs undergoing surgical castration on d 1 and d 2 postcastration, but no mean difference in HR, RR, or rectal temperature between treatment groups following surgical castration regardless of lidocaine or flunixin usage. Continuous HR monitors would be useful to determine if human interactions contributed to the HR and RR measured.

### Serum Cortisol and PGE_2_

There were no significant effects of treatment, time, or their interaction on mean serum cortisol (*P* > 0.3) or PGE_2_ (*P* > 0.2) (Table 3). FM CAST goats did have a numerically lower mean cortisol value than PL CAST at 12 h despite no statistical difference (FM CAST-5.3033 ± 0.324 ng/mL; PL CAST-5.6013 ± 0.324 ng/mL); however, this trend was not present at 24 or 96 h. According to recent research, calves receiving TD FM at the time of castration have lower mean cortisol levels compared with castrated placebo-treated animals at 2, 3, and 4 h postcastration (Kleinhenz et al., 2018b). The aforementioned study did not employ the use of a local anesthetic as in this study. Cortisol responses were significantly reduced when utilizing local lidocaine or bupivacaine, which may be one reason no difference was seen between treatment groups in the current study (Earley and Crowe, 2002; Stafford et al., 2002; Coetzee, 2012). That said, local anesthetics alone with surgical castration do not appear to reduce the cortisol response; however, the use of local lidocaine along with NSAIDs has been shown to be effective at eliminating the cortisol response as seen in this study where no statistical difference was noted between treatment groups (Fisher et al., 1996; Stafford et al., 2002). Additionally, the authors speculate that differences in cortisol and PGE_2_ were not detected due to sample time point collection. Samples in the current study were only taken at 0, 12, 24, 72, and 96 h, which therefore may have allowed the researchers to miss the early differences in cortisol and PGE_2_ seen in other studies.

The lack of significant effects of treatment, time, or their interaction on mean serum PGE_2_ (*P* > 0.2) is contrary to findings of other published reports in goats which demonstrated a decrease in PGE_2_ following TD FM administration (Reppert et al., 2019a). However, unlike the current study, an ex vivo model was utilized in which lipopolysaccharide from Escherichia coli was added to samples, incubated, and centrifuged before determining the PGE_2_ concentration for each time point. Limited sample collection time points prior to 12 h may have caused the researchers to miss a reduction in PGE_2_ as Fraccaro et al. (2013) only found significant reduction of PGE_2_ following dehorning in calves for up to 12 h when flunixin was administered intravenously. An additional explanation for the lack of PGE_2_ reduction in the current study may also be the lower bioavailability of TD FM in goats (24.76%) as reported by Reppert et al. (2019). Lack of PGE_2_ suppression may also be associated with age as demonstrated in calves, perhaps indicating a greater half maximal inhibitory concentration for young goats (Kleinhenz et al., 2018a). Further research with greater sample sizes and more frequent sample collection are warranted to determine varying flunixin effects on PGE_2_ following painful stimuli in an in vivo model.

Pain mitigation associated with castration is an important animal welfare concern for all involved in this industry, which will continue to gain in prominence as the production of goat meat within the United States continues to expand. In this study, TD FM application demonstrated a similar mean concentration and therapeutic window compared to intravenous flunixin, establishing TD application as an effective delivery method for young goats. Goats treated with TD FM had greater feed intake following castration, but this was insufficient to alleviate weight loss. Still, increased appetite suggests a positive outcome from the use of TD FM. No differences were observed in measures of acute pain and inflammation when compared to placebo treated control goats. Future research is necessary to establish an appropriate TD FM administration protocol or multimodal analgesic model to effectively provide adequate pain relief that is both practical and cost effective.
